# Effects of Steroids and Curcumin on Prevention of Laryngeal Stenosis Secondary to Trauma

**Published:** 2016-07

**Authors:** Kamyar Iravani, Zahra Babaie, Mohammad-Javad Ashraf, Nader Tanideh

**Affiliations:** 1 *Department of Otorhinolaryngology, Shiraz University of Medical Sciences, Shiraz, Iran.*; 2 *Department of Pathology, Shiraz University of Medical Sciences, Shiraz, Iran.*; 3 *Stem cell and Transgenic Technology Research Center, Shiraz University of Medical Sciences, Shiraz, Iran.*

**Keywords:** Corticosteroids, Curcumin, Laryngeal stenosis, Laryngeal scar

## Abstract

**Introduction:**

The aim of this study was to compare the preventive effects of corticosteroids and curcumin on subglottic stenosis in an animal model.

**Materials and Methods:**

Twenty-one male German Shepherd dogs were used for this study. After standardized trauma to the subglottic area, the dogs were divided into three groups. Group A received curcumin (450 mg/ day) for 15 days; Group B received beclomethasone (2 puffs/day, 50 µg/dose) for 15 days; Group C received saline spray only. At 6 weeks after the injury, the larynx specimens were examined histopathologically to assess epithelialization, inflammation, and fibrosis.

**Results:**

Complete epithelial covering of the steroid-treated group was significantly less than that of the control group. Despite inflammation and fibrosis, there was no significant difference between the steroid and control groups. In the curcumin-treated group, there was no significant difference between the groups.

**Conclusion:**

Topically applied steroid decreases epithelialization after induced subglottic injury. It is recommended that further studies be conducted in order to investigate the effects of the two drugs on airway stenosis prevention.

## Introduction

Laryngotracheal stenosis is a devastating, challenging, and sometimes life-threatening condition. This type of stenosis requires a multidisciplinary approach with multiple treatment options and variable outcomes. It seriously affects the quality of life in patients, despite multiple surgical procedures. 

There are many causes of laryngotracheal stenosis including prolonged endotracheal intubation, laryngeal procedures, external trauma, high tracheotomy, radiation therapy, and thermal and chemical burns (1,2).

Optimal treatment modalities for subglottic and upper tracheal stenosis depend on the severity of the stenosis and the general condition of the patient. The major problem for each modality, including the endoscopic and open reconstructive approach, is scar formation and restenosis (1-3). Moreover, fibroblasts and other inflammatory cells have a key role in scar formation and fibrosis (3,4). 

Multiple agents have been used in an attempt to reduce scar formation in experimental and clinical situations after laryngeal injury, including systemic and topical steroids, antibiotics, mitomycin C, heparin, vitamin A, and 5-fluorouracil (5-FU). However, the precise effects of these agents have not yet been demonstrated (1-3).

Corticosteroids have antiinflammatory and immune-suppressive effects and are frequently used in immunologic, hematologic, and inflammatory diseases. They inhibit migration of inflammatory cells, inactivate tissue macrophages, and reduce inflammatory mediators such as interleukin-1, tumor necrosis factor alpha (TNF-α), cyclooxygenase-2 and prostaglan- dins (5-7). In the airways, steroids have a dual effect. They reduce edema, decrease inflammation, and inhibit collagen synthesis, resulting in the prevention of scar formation in the early stage of wound healing (8,9). On the other hand, they are believed to have a harmful effect on wounds by causing delay in healing (10-12). Curcumin is the most active component of the turmeric (Curcuma longa) plant. The predominant substance in curcumin is diferuloylmethane (C_2_H_2_0O_6_) (13,14). Curcumin is considered to have antiinflammatory, anti-oxidant, pro-apoptosis, and cancer preventive and treatment effects, according to many experimental and clinical investigations (15). Some investigations have shown that curcumin inhibits inflammatory mediators such as nuclear factor-kappa B (NF-kB), cyclin D1, cyclooxygenase (COX-2), interleukin-6 and interleukin-8 (16-19).

The purpose of this experimental study was to evaluate the efficacy of corticosteroids and curcumin in laryngeal scar prevention in an experimental dog model for future clinical applications.

## Materials and Methods

Twenty-one male German Shepherd cross-bred dogs, weighing between 25 and 30 kg, were used in this study. The animals were randomly allocated to three groups; Group A (curcumin group), Group B (corticosteroid group), or Group C (control group), with seven dogs in each group. After general anesthesia was administered by intramuscular injections of xylazine (0.15 mg/kg) and ketamine(10 mg/kg), the dogs were placed in the supine position.

Visualization of the larynx was achieved with a long-blade laryngoscope and through an operating microscope with a focal point of 400. Chemical injury was performed using a ball of cotton soaked in hydrochloric acid (30% concentration) that was positioned in the subglottic area while maintaing contact with the mucosa for 10 s. Next, physical injury was achieved using a long hook that traumatized the subglottic mucosa and the underlying cartilage circumferentially. 

All animals received antibiotic (penicillin, 3×10^6^ unit/day) for 3 days to prevent infection that may have impaired the healing process. Group A dogs received curcumin spray (450 mg/day) under visualization with laryngoscope and general anesthesia for 15 days. Group B dogs received two puffs of beclomethasone dipropionate inhalation spray (50 µg/dose) daily for 15 days using the same method as described for Group A. Group C received saline spray only. After 6 weeks all the dogs were killed by KCl and the larynx of each dog was harvested and fixed with 10% buffered formalin solution. The specimens were investigated for any gross and microscopic changes.

Appropriate full-thickness tissue samples were taken from the subglottic area. Tissue samples underwent routine histologic processing. 5-micrometer histologic sections were prepared and stained with hematoxylin and eosin (H&E). The sections were examined under a light microscope using an ocular micrometer for determining the thickness of the fibrous tissue.

 Epithelial covering, degree, and type of inflammatory cell infiltration and collagen deposition were graded according to the methodology of Cincik et al. (3). All analyses were performed using SPSS statistical software, version 18.0 (SPSS Inc., Chicago, IL).

Statistical significance was set at an alpha of 0/05 (or P<0.05). Fisher’s exact test and the Mann-Whitney test were used as appropriate. Tables 1, 2, and 3 show the grading systems applied.

**Table 1:** Grading of epithelial covering based on percentage of mucosal surface covered by epithelium

**Epithelial covering**	**<25%**	**25–50%**	**50–75%**	**>75%**
Grade	1	2	3	4

**Table 2:** Grading of inflammation based on number of inflammatory cells per (×10) microscope fieldNo. inflammatory cells per ×10 field

	**0–10**	**11–50**	**51–100**	**>100**
Inflammation grade	1	2	3	4

**Table 3:** Grading of fibrosis based on thickness of subepithelial fibrous tissue in micrometerThickness

	**<400 µ**	**400–800 µ**	**>800 µ**
Fibrosis grade	Mild	Moderate	Severe

## Results

Grades of the epithelial covering, inflammation, and fibrosis of the three groups are summarized in Tables 4, 5, and 6, respectively. Epithelial covering in Group 1 (curcumin) was complete (Grade 4) in all seven cases, while in Group 2 (steroid), only three cases (42.9%) showed complete epithelialization (Grade 4) and the other four cases (57.1%) showed Grade 3 epithelial covering. The difference between these two groups was statistically significant(P=0. 019). The control group also showed complete epithelialization in all seven cases (Table. 4).

**Table 4:** Number and percentage of epithelial grades for each group

**Group**	**Epithelial grade**		
	Total	4 No. (%)	3 No. (%)
1	0 (0)*	7 (100)*	7
2	4 (57.1)*	3 (42.9)*	7
3	0 (0)	7 (100)	7
Total	4 (19)	17 (81)	21

* 2 groups with statistical significance

The grades of inflammation in the three groups showed no statistically significant difference. Three out of seven cases in Group 1 (curcumin) showed Grade 4 inflammation, while the other two groups (steroid and control) showed more cases with Grade 2 or 3 inflammation. Regarding the type of inflammatory cells, although there was no statistically significant difference between the groups, more cases (five out of seven) in Group 1 (curcumin) exhibited acute inflammatory cells as well as chronic cells. In Group 2 (steroids), most cases (six out of seven) showed only chronic inflammatory cells. The control group also revealed mixed acute and chronic inflammatory cells (Table.5).

**Table 5:** Number of cases in different inflammation grades for each group

**Group**	**Inflammation grade**						
	No. 1	No. 2	No. 3	No. 4	Mixed No.	Chronic No.	Total
1	1	2	1	3	5	2	7
2	2	2	2	1	1	6	7
3	2	3	1	1	3	4	7
Total	5	7	4	5	9	12	21

**Fig 1:**
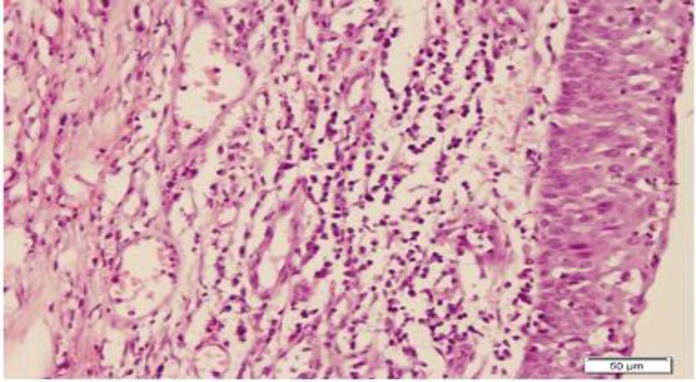
Complete epithelialization with mixed acute and chronic inflammation in case of curcumin inhalation, H&E stain (×200

**Fig 2:**
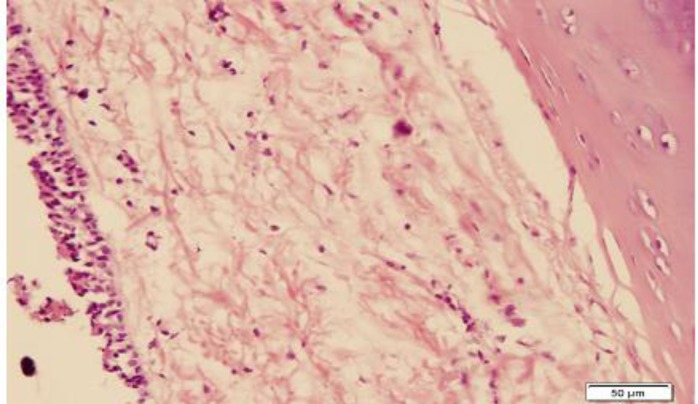
Mild chronic inflammation and mild fibrosis in case of steroid inhalation, H&E stain (×200

**Fig 3:**
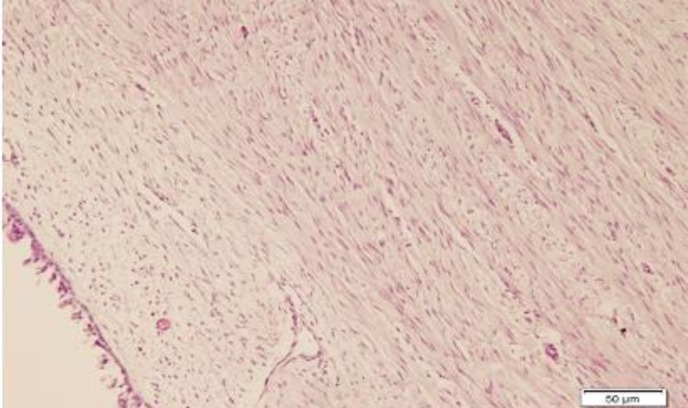
Moderate-to-severe fibrosis in case of control group, H&E stain (×100

The grades of fibrosis in the three groups showed no statistically significant difference. More cases (four out of seven) in Group 2 (steroid) showed mild fibrosis, while most of the cases in the control group showed moderate-to-severe fibrosis. In Group 1 (curcumin), the degree of fibrosis showed no trend, as summarized in Table 6. Figures 1, 2, and 3 show the histologic differences in the three groups.

**Table 6:** Number of cases in different grades of fibrosis for each group

**Group**	**Fibrosis grade**			
	Mild No.	Moderate No.	Severe No.	Total
1	2	3	2	7
2	4	2	1	7
3	0	5	2	7
Total	6	10	5	21

## Discussion

Many agents have been used in an attempt to reduce stenosis in the laryngotracheal airway in experimental and clinical models. We used steroid and curcumin based on their previously reported effects on inflammation and wound healing in various organs. Cinicik et al. used mitomycin C and 5-FU/triamcinolone on scar formation on the subglottic area in a rabbit model. Their study showed significantly decreased fibrosis in rabbits treated with these two agents with complete epithelial lining (3).

Talas et al. studied the effects of corticosteroids and vitamin A on the healing of tracheal anastomoses in a rat model. They concluded that steroids may have a delaying effect on wound healing steps, including epithelial regeneration; this is similar to our findings in the steroid group (10). Ingrams et al. used a combination of 5-FU and triamcinolone to reduce the subglottic stenosis in a rabbit model. They noticed increased chronic inflammation and fibrosis formation in the control group compared with the treated group. They also detected complete epithelialization in the treated group (20), which may be due to the combined application of 5-FU and triamcinolone. Ertugrul et al. used halofuginone, an antimetabolic agent, in the management of the subglottic stenosis in a rat model. They detected decreased fibrosis with complete epithelial regeneration in the treated group (1). Yoon et al. also successfully used halofuginone on the posterior glottic stenosis in a rabbit model. Their study showed less scar and granulation tissue formation in the treated group (21). Furthermore, Roh used topical mitomycin C in a rabbit model after laryngeal injury by laser. He reported less scarring, granulation formation, and synechia of the injured posterior glottis (22).

Data from the present study reveal no significant difference between the groups according to inflammatory cells and fibrosis criteria. This may be due to the low sample size of the groups in this study. However, for epithelial covering, steroid-treated animals significantly differed from the curcumin and control groups. Although we used the typical dose of steroids, the steroid-treated group showed delay in epithelialization. This finding shows that steroids may have an adverse effect on the healing process.

## Conclusion

Traditionally, curcumin and steroids are believed to be antiinflammatory agents with a positive effect on wound healing. To the best of our knowledge, no study has yet compared the efficacy of these drugs in decreasing scar and fibrosis after subglottic trauma in a canine model. Our findings show that curcumin has no effect on scar prevention in the airways. Steroids also have a delaying effect for epithelialization and healing in the airway. Further studies are required to investigate the efficacy of these drugs.
